# Experience with periprosthetic infection after limb salvage surgery for patients with osteosarcoma

**DOI:** 10.1186/s13018-021-02243-6

**Published:** 2021-01-28

**Authors:** Tiao Lin, Qinglin Jin, Xiaolin Mo, Zhiqiang Zhao, Xianbiao Xie, Changye Zou, Gang Huang, Junqiang Yin, Jingnan Shen

**Affiliations:** 1 Department of Musculoskeletal Oncology Center, The First Affiliated Hospital of Sun Yat-sen University, 58 Zhongshan 2nd Rd, Guangzhou, 510080 China; 2 Guangdong Provincial Key Laboratory of Orthopedics and Traumatology, Guangzhou, 510080 China; 3 Zhongshan School of Medicine, Sun Yat-sen University, Guangzhou, China

**Keywords:** Osteosarcoma, Periprosthetic joint infection, Limb salvage surgery, Revision, Risk factor

## Abstract

**Background:**

The rate of postoperative infection developing is higher after limb salvage surgery (LSS) following sarcoma resection compared with conventional arthroplasty. The goal of this study is to summarize our experience in management of periprosthetic joint infection (PJI) and the risk factors of early PJI after LSS.

**Methods:**

Between January 2010 and July 2019, 53 patients with osteosarcoma in the lower extremities who encountered periprosthetic infection after segmental tumor endoprosthetic replacement in our center were analyzed. Detailed patient characteristics and therapeutic information were collected from database of our institution or follow-up data and we divided patients according to the interval time between infection and tumor resection (surgery-infection interval) and investigate potential risk factors.

**Results:**

A total of 53 (5.08%) patients were suffered postoperative infection. The average interval between surgery and clinical signs of deep infections are 27.5 days. For the drainage culture, positive results were only presented in 11 patients (20.8%). Almost half of this study’s (47.2%) patients underwent a traditional two-stage revision, that was, after the removal of the infected prosthesis, we applied antibiotic-loaded bone cements as a spacer. The mean blood loss during initial implantation surgery and operation time both correlated with interval period between PJI and initial implantation significantly (*P* = 0.028, *P* = 0.046). For several patients which infection marker was hardly back to normal after spacer implantation, we conservatively introduced an improved combination of bone cement and prosthesis for the second-stage surgery (5.6%). There were six patients needing re-operation, of which three were due to the aseptic loosening of the prosthesis, one developed periprosthetic infection again, and two patients encountered local recurrence and underwent amputation. Two patients were dead from distal metastasis.

**Conclusions:**

A two-stage revision strategy remains effective and standardized methods for PJI patients. Total operation time and blood loss during LSS of osteosarcoma are the main risk factors of early PJI. For the patients without confirmed eradiation of microorganisms, an improved combination of bone cement and prosthesis applied in the second-stage surgery could achieve satisfied functional and oncologic results.

## Introduction

With advances in neoadjuvant and adjuvant chemotherapy, the long-term survival rate of patients with osteosarcoma has increased to almost 70% [1, 2]. As such, more than 95% of patients with osteosarcoma of lower extremities are candidates for a limb salvage surgery (LSS) [3, 4], in which an endoprosthesis is used for reconstruction after tumor resection.

Compared with traditional arthroplasty of the lower limb, the rate of periprosthetic joint infection (PJI) is markedly higher in patients who undergo LSS, with a reported incidence of 8% to 19.5% [5–9]. Infection can result in the removal of the prosthesis and a delay in administering chemotherapy. Risk factors for PJI include a poorer condition of the soft tissue, a greater number of cycles of chemotherapy, longer length of bone resection, longer operation time, and the size of the primary tumor [5, 7, 10].

An early diagnosis of PJI is critical to achieving a good outcome; however, diagnosis can be difficult and requires multidisciplinary cooperation. In addition to symptoms and signs, serological examinations including white blood cell (WBC) count, erythrocyte sedimentation rate (ESR), C-reactive protein (CRP), and blood and tissue cultures can assist in the diagnosis [11, 12]. In addition, the occurrence of PJI may be related to the virulence of the microorganism, while the positive culture rate is not high [13–15]. The heterogeneity inherent with PJI also leads to difficulties in treatment, and challenges include local infection control, limb function preservation, and length of hospital stay reduction [16, 17].

The purpose of this report was to summarize our experience in management of PJI and the risk factors of early PJI after LSS.

## Methods

### Study population

Between January 2010 and July 2019, 53 patients with osteosarcoma of the lower extremities who received LSS at our center and developed a PJI were included in the analysis. All patients were diagnosed with PJI based on an algorithm and an interdisciplinary team of surgeons, microbiologists, pathologists, and infectious disease specialists [15].

Detailed patient characteristics and treatment information were collected from the medical records, including demographic data (age, sex), tumor site, tumor histological type, operation time, blood loss, drainage culture results, and functional and oncological data. Functional assessment was based on the musculoskeletal tumor society (MSTS) scoring system. Influential factors that contributed to the time after surgery PJI was diagnosed were also examined.

### Method of classification

PJI is typically manifested as an acute onset of joint pain, effusion, erythema and warmth at the implant site, and fever and are commonly caused by virulent microorganisms. Diagnosis of PJI is according to the clinical manifestation, laboratory examination, and radiological examination (加个参考文献). Patients were divided into three groups based on the interval between surgery and diagnosis of infection: early, PJI was diagnosed within 2 months of surgery; delayed, PJI was diagnosed 3 to 24 months after surgery; and late, PJI was diagnosed more than 24 months after surgery [18, 19]. The Institutional Ethical Board of our hospital approved this study.

### Treatment of infection

**Fig. 1:**
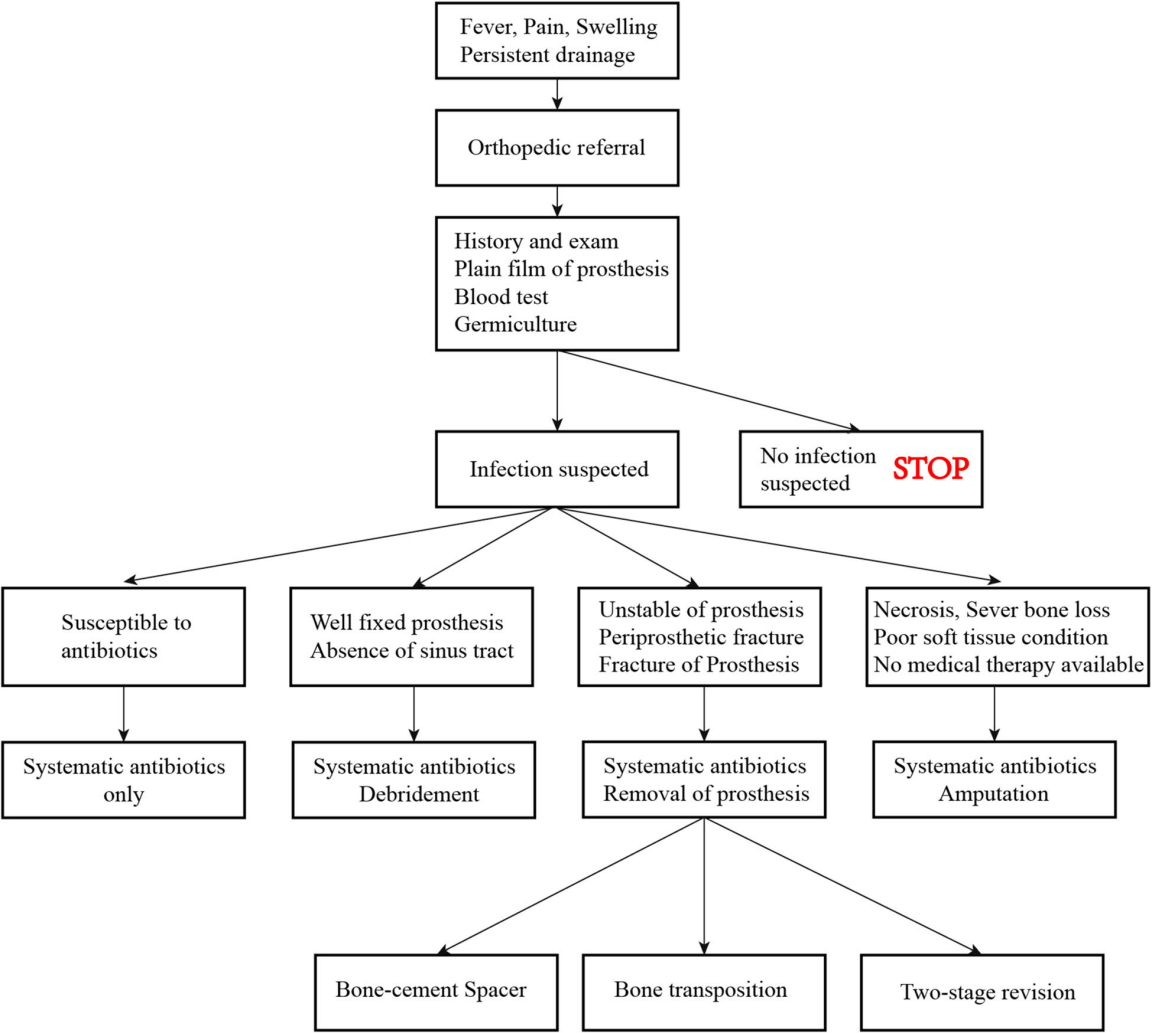
Management of periendoprosthetic joint infection

Based on PJI treatment guidelines, seven treatment strategies were used: (1) systemic antibiotics, (2) debridement plus irrigation, (3) bone-cement spacer placement, (4) traditional two-stage prosthesis revision, (5) bone transposition, (6) combined implantation of cement and prosthesis, and (7) amputation (Fig. 1) [16]. In addition, we treated some patients with a new reconstruction method using a customized prosthesis combined with antibiotic-loaded cement surrounding the stem during the second-stage surgery.

### Statistical analysis

Categorical variables were expressed as numbers and percentages, and the χ^2^ test was used to compare differences between groups. Continuous variables were presented as the mean (range), and means were compared by the test. SPSS version 24.0 statistical software (IBM Corporation, New York, USA) was used for all statistical analyses. A value of *P* < 0.05 was considered statistically significant.

## Results

### Summary of patient characteristics and treatments

The mean age at the time of diagnosis of the 53 patients was 20 years (range 9–48 years). There were 37 males (69.8%), and the median follow-up time was 46 months (range 12–91 months). Primary tumor sites were the distal femur (*n* = 27, 50.9%), proximal tibia (*n* = 24, 45.3%), and proximal femur (*n* = 2, 3.8%). The mean blood loss of the primary surgery was 455.7 mL (range 100–2800 mL), and the average time was 4.8 h (range 1.5–12 h) (Table 1).

**Table 1:** Characteristics of the patients

Characteristics	Information
Age, years	20.0 ± 8.4
Gender, *N* (%)	
Male	37 (69.8)
Female	16 (30.2)
Tumor site, *N* (%)	
P. Femur	2 (3.8)
D. Femur	27 (50.9)
P. Tibia	24 (45.3)
Initial surgery	
Blood loss, mean ± sd (range) mL	497.8 ± 477.5 (100–2800)
Operation time, mean ± sd (range) hours	4.8 ± 2.03 (1.5–12)
Time to infection post-op, mean ± sd (range) months	20.1 ± 29.3 (1–121)
Interval between spacer insertion and second revision, mean ± sd (range) months	6.9 ± 5.3 (4–18)
Pre- and post-operation chemotherapy, *N* (%)	53 (100)
Infectious manifestation, *N* (%)	
Fever	15 (28.3)
Localized redness, swelling, heat and pain	28 (52.8)
Malodorous drainage	26 (49.1)
Germiculture, *N* (%)	
Staphylococcus aureus	5 (9.4)
Staphylococcus epidermidis	3 (5.7)
Escherichia coli	1 (1.9)
Stenotrophomonas maltophilia	1 (1.9)
Pseudomonas aeruginosa	1 (1.9)
None	38 (79.2)

*P*. *Femur* proximal femur, *D*. *Femur* distal femur, *P*. *Tibia* proximal tibia

The mean time of PJI diagnosis after the primary surgery was 20.1 months (range 1–121 months), and the average interval between spacer insertion and second revision was 6.9 months (range 4–18 months). The mean time interval time between the appearance of PJI (swelling, redness, pain and malodorous drainage, etc.) and the initial implantation was 27.5 days. Localized redness, swelling, heat, and pain were observed in 28 patients (52.8%), malodorous drainage occurred in 26 patients (49.1%), and fever only was seen in 15 patients. Only 11 patients (20.8%) had positive in germicultures of blood or tissue (5 *Staphylococcus aureus*, 3 *Staphylococcus epidermidis*, 1 *Escherichia coli*, 1 *Stenotrophomonas maltophilia*, 1 *Pseudomonas aeruginosa*) (Table 1).

**Fig. 2:**
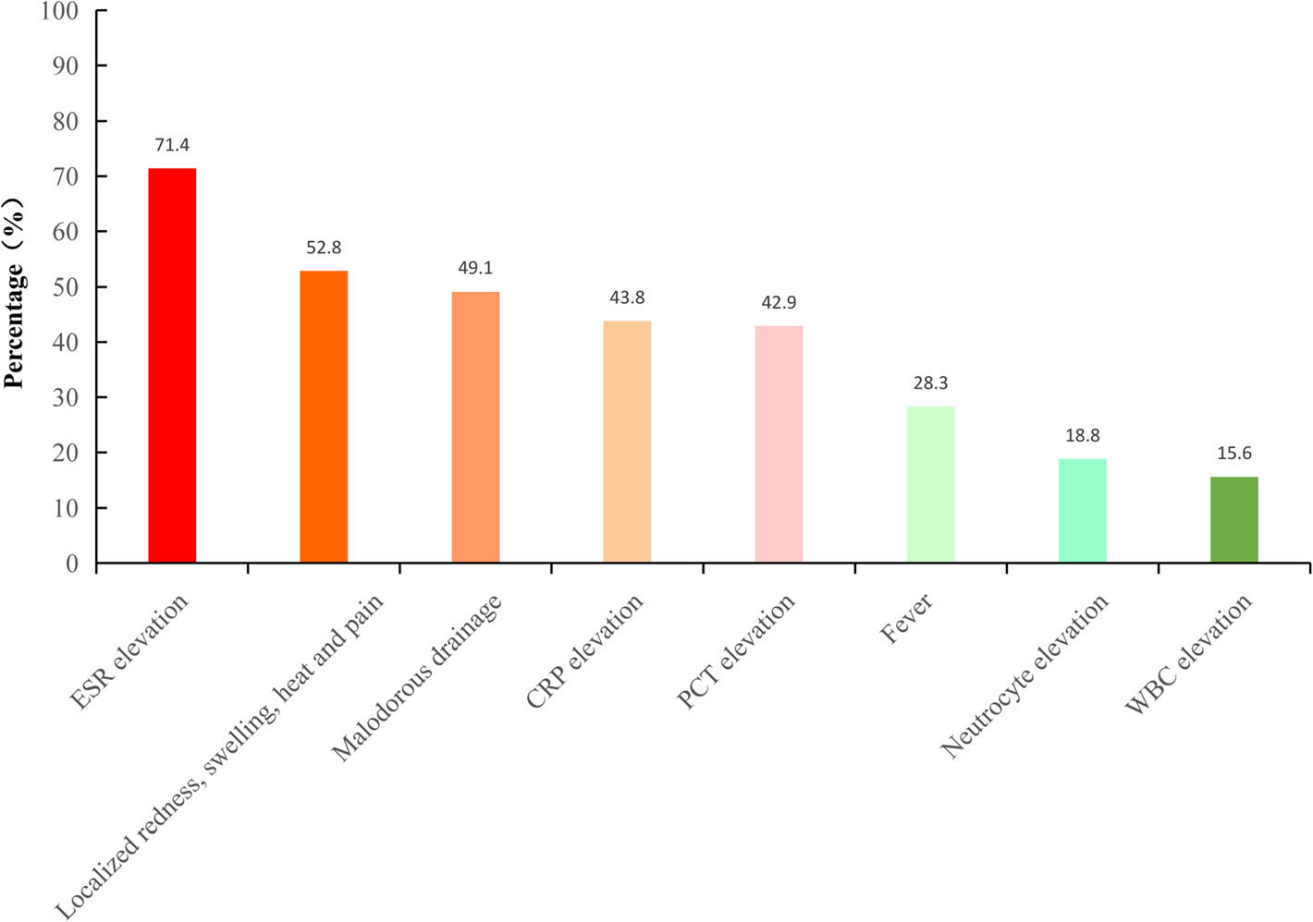
Early diagnostic indication of periendoprosthetic joint infection

We examined signs, symptoms, and several conventional inflammatory markers to identify the most sensitive indicator of PJI. Of the 53 patients, 71.4% had an elevated ESR while only 43.8% had an elevated CRP level and 42.9% had an elevated procalcitonin (PCT) level. Only 28.3% patients developed a fever, and only 18.8% of patients had an elevated neutrophil count (Fig. 2).

The infection was successful treated with systemic antibiotics only in 3.8% of patients, debridement and irrigation in 9.4%, and bone-cement spacer replacement without second-stage revision in 15.2%. Only 1 patient (1.9%) received bone transposition, and most patients (47.2%) received traditional 2-stage prosthesis revision with good outcomes (Table 2). However, 9 patients (16.9%) required an amputation. Six patients required a re-operation, of which 3 had aseptic loosening of the prosthesis, 1 developed a second peri-prosthetic infection, and 2 developed a local recurrence and underwent amputation. Two patients died from distal metastasis. Of the surviving patients, the functional results were satisfactory with a mean MSTS score of 20 (range 17–23).

**Table 2:** Successful modality

Types	*N* (%)
Systemic antibiotics	2 (3.8)
Debridement and irrigation	5 (9.4)
Bone-cement spacer placement	8 (15.2)
2nd prosthesis revision	25 (47.2)
Amputation	9 (16.9)
Bone transposition	1 (1.9)
Combined prosthetic revision with cement	3 (5.6)
Total	53 (100)

### Comparison of potential risk factors for PJI at different times after the primary surgery

**Fig. 3:**
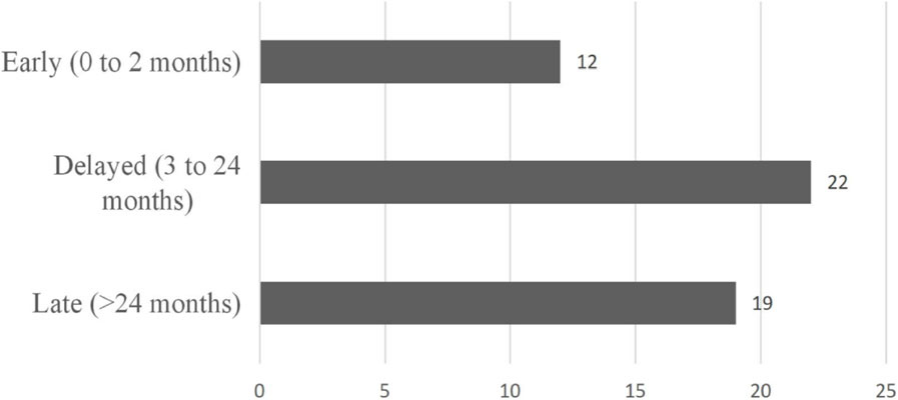
Time to infection from initial implantation

Twelve patients (22.6%) had an early PJI infection, 22 (64.2%) a delayed infection, and 19 (13.2%) a late infection (Fig. 3). The mean blood loss during the primary surgery was 791 ± 705 mL (range 300–2800 mL) in the early group, 358 ± 200 mL (range 100–800 mL) in the delayed group, 317 ± 138 mL (range 100–600 mL) in the delayed group (*P* = 0.028). The mean operation time of the primary surgery in the early group was 7.4 ± 2.5 h (range 5–12 h), in the delayed group was 4.3 ± 1.3 h (range 2–8 h), and in the late group was 3.4 ± 0.8 h (range 2–4.5 h) (*P* = 0.046). Furthermore, 3 patients (25%) received expanding surgery after the initial implantation in the early group, while only 2 patients (9.1%) and 1 patient (5.3%) received expanding surgery in the delayed and late group, respectively (*P* = 0.047). In the early group, 5 patients (41.7%) had positive cultures at the time of PJI diagnosis, while 4 patients (18.2%) in the delayed group had positive cultures, and 2 patients (10.5%) in the late group had positive cultures (*P* = 0.044) (Table 3). These results suggest that substantial blood loss during the primary surgery, prolonged operation time, and expanding surgery after initial implantation might increase the risk of early PJI.

**Table 3:** Risk factors of PJI in different period after implantation

Items	Classification of infection time to post-op			*P* value
	Early	Delayed	Late	
Blood loss	791 ± 705	358 ± 200	317 ± 138	0.028
Mean ± SD (range) mL	(300, 2800)	(100, 800)	(100, 600)	
Operation time	7.4 ± 2.5	4.3 ± 1.3	3.4 ± 0.8	0.046
Mean ± SD (range) hours	(5, 12)	(2, 8)	(2, 4.5)	
Tumor site *N* (%)				
Distal femur	6 (50)	10 (45.5)	11 (57.9)	> 0.05
Proximal tibia	6 (50)	12 (54.5)	8 (42.1)	
Expandation *N* (%)	3 (25.0)	2 (9.1)	1 (5.3)	0.047
Chemotherapy *N* (%)	12 (100)	22 (100)	19 (100)	> 0.05
Germiculture *N* (%)	5 (41.7)	4 (18.2)	2 (10.5)	0.044
ESR *N* (%)				
High	9 (75.0)	15 (68.2)	14 (73.7)	> 0.05
Normal	3 (25.0)	7 (31.8)	5 (26.3	
CRP *N* (%)				
High	6 (50)	9 (40.9)	11 (57.9)	> 0.05
Normal	6 (50)	13 (59.1)	8 (42.1)	

*ESR* erythrocyte sedimentation rate, *CRP* C-reactive protein

### An improved treatment strategy for treatment of PJI

**Fig. 4:**
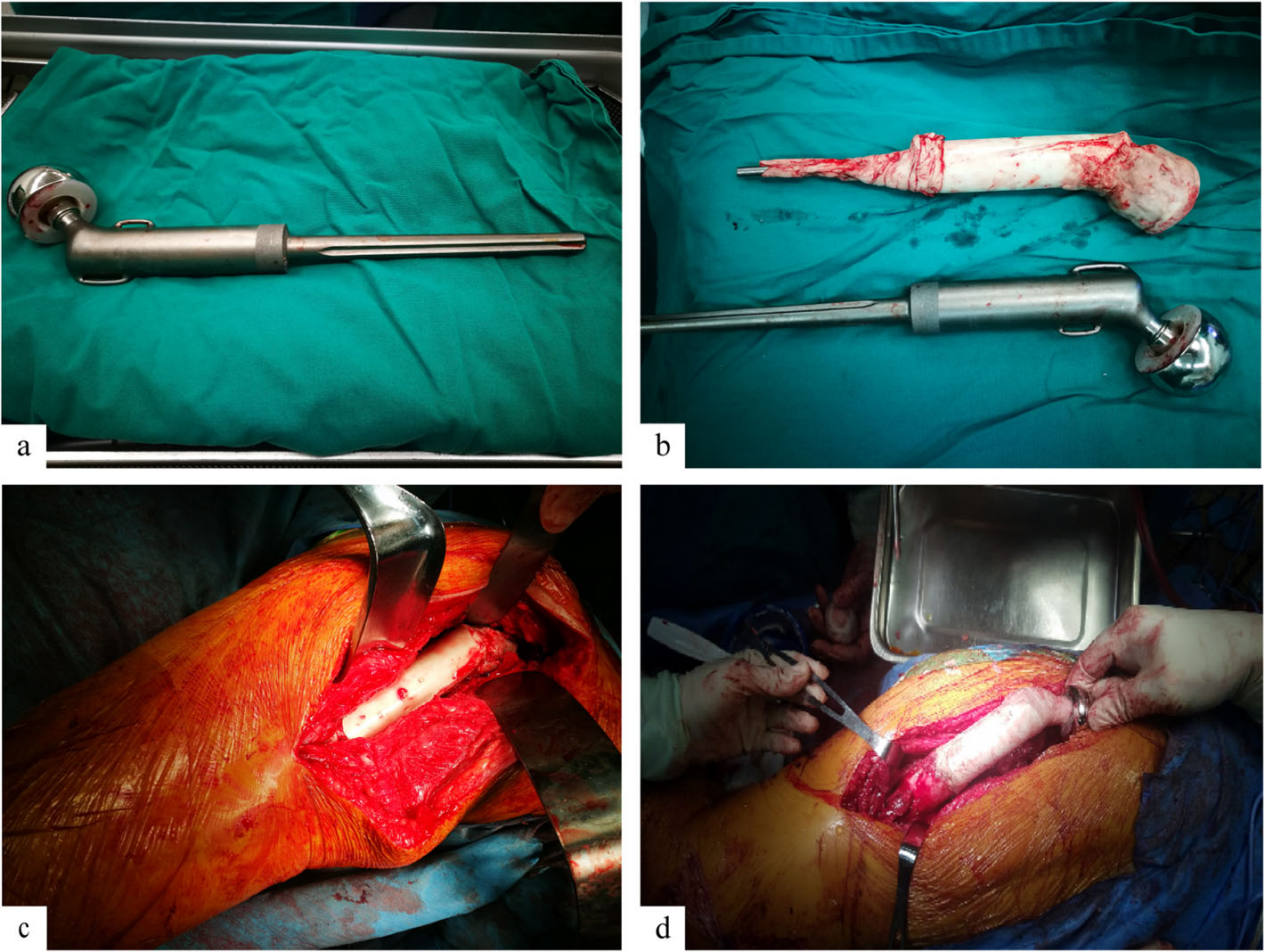
The improved two-stage revision. Removed endoprosthesis at the first stage of revision (**a**). Cement-decorated spacer for the first stage of revision (**b**). Implantation of cement-decorated spacer at the first stage of revision (**c**). Implantation of endoprosthesis with cement decorating at the second stage of revision (**d**)

An improved strategy of combined prosthetic revision with cement was used in 3 (5.6%) patients without any complications (Fig. 4).

**Fig. 5:**
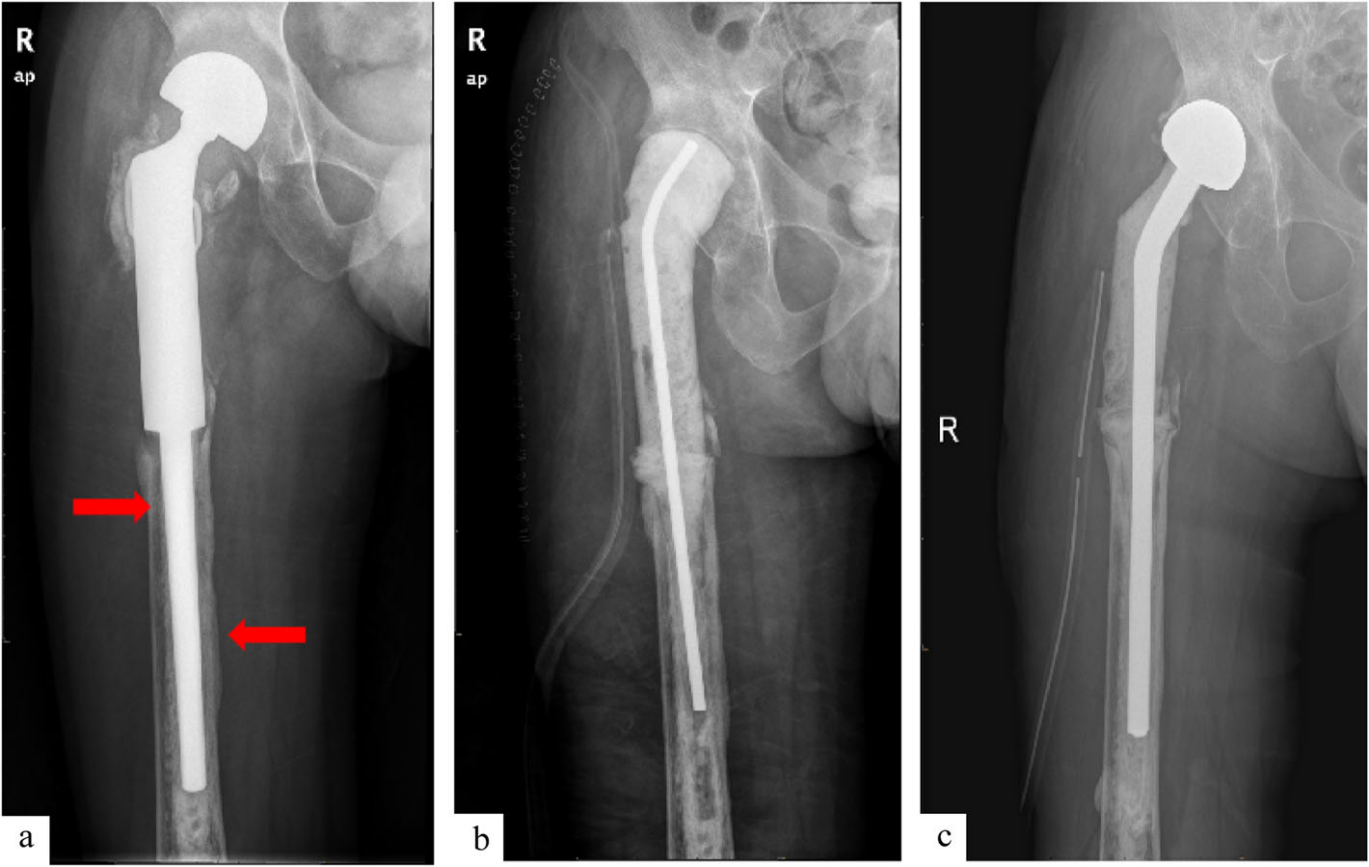
X-ray image of the patients undertaken the improved two-stage revision. Posterior-anterior image of the patient diagnosed of PJI (**a**). Posterior-anterior image of the patient after the first-stage of revision (**b**). Posterior-anterior image of the patient after the second-stage of revision (**c**). # Red arrow means lesion of bone absorption

As a representative case, a 33-year-old male had a tumor resection and implantation of a hip joint 8 years prior was seen with localized swelling and worsening hip pain for 6 months. Radiographs indicated peri-prosthetic bone loss. Laboratory studies showed elevated ESR and CRP levels, normal PCT and WBC count, and negative cultures before and after surgery. The patient received a 2-stage revision surgery with the second stage consisting of prosthesis implantation with cement (Fig. 5). The revision was completed within 1 year, and he was walking unaided 4 months after the second stage procedure. At the last follow-up, the patient was satisfied with the outcome, and his MSTS score was 22 without any recurrence of infection or tumor.

Therefore, this improved surgical strategy for PJI well balance local infection control and limb functional preservation.

## Discussion

LSS with endoprosthesis implantation is currently recommended for patients with osteosarcoma of the lower limbs [3–5]. However, the procedure is associated with a high risk of PJI, with an incidence markedly greater as compared with conventional knee and hip joint arthroplasties [5, 6, 8–10]. In this study, the overall rate of PJI was 5.08% (53/1044), which is in accordance with the rates reported in other studies, and most patients had delayed (22/53, 41.5%) or late (19/53, 35.8%) infections [15].

As positive cultures are not present in many patients with a PJI, the diagnosis is primarily made by considering medical history, physical examination findings, and laboratory studies of inflammatory factors [13, 20, 21]. It is important to note that early detection and treatment is critical for achieving good outcomes in patients with a PJI. In this study, we observed that the ESR was elevated early in the course of the disease in 71.7% of the patients, while CRP and PCT were only increased 43.8% and 42.9%, respectively, or patients. With respect to signs and symptoms, overall 52.8% of patients experienced localized redness, swelling, heat, and pain of the affected limb, and 49.1% patients had malodorous drainage at the time of diagnosis. Because culture results were only positive in 20.8% of the cases, combining clinical symptoms and signs with ESR might be more dependable for a diagnosis of PJI at an early stage.

Tumor volume, the site of the tumor, chemotherapy, hematoma, inadequate soft tissue coverage, and operation time have all been described as associated with the occurrence of PJI [5, 7, 17]. However, few studies have reported risk factors for early or late PJI after the primary surgery. In this study, we observed that substantial intra-operative blood loss was associated with early PJI (*P* = 0.028) (Table 3). To some extent, prolonged operation time is a proxy for the complexity of the surgical procedure. We also found that the volume of intra-operative blood loss during the primary surgery was a risk factor for PJI within 2 months of the surgery (*P* = 0.028) (Table 3). For immunocompromised patients who received neoadjuvant and adjuvant chemotherapy, a large volume of blood loss during surgery can further disrupt the immune system resulting in early PJI after surgery [17, 22].

In the early infection group, three (3/12, 25%) patients received expanding surgery after the initial implantation, and this number was two (2/22, 9.1%) in the delayed group and one (1/19, 5.3%) in the late group (*P* = 0.044) (Table 3). Other studies have reported that an endoprosthesis lengthening procedure increases the risk of PJI of up to 5% [14, 15, 22, 23].

For patients with a PJI, the re-infection rate is increased dramatically, and successful local infection control is critical for these patients [24–27]. It is therefore critical to formulate suitable treatment strategies in consultation with a multidisciplinary team for combined surgical and medical management. Currently, two-stage revision with complete removal of the endoprosthesis is the primary treatment for these patients [25–27]. In our study, more than half of all patients received two-stage revision, among which three patients were treated with combined prosthetic revision and implantation with cement at the second stage of the revision (Fig. 4). All patients who received a two-stage revision recovered without any complications. However, nine patients (16.9%) required amputation for reasons including uncontrolled infection and re-infection, and other reasons such as economic status and personal requirements (Table 2). Amputation as a last choice for PJI was performed for late uncontrolled infections with systemic signs, and this is one of the most effective treatments for PJI. However, it is hard to accept amputation for most of young patients with an average age of 20 years old

In this study, we introduced an improved revision strategy in which the second stage is prosthetic revision combined with cement (Fig. 4). In the case presented, the patient was able to walk unaided 4 months after the second-stage procedure, and his MSTS score was 22 at the last follow-up. Gundavda et al. [26] demonstrated that the mean MSTS score of patients with PJI after initial implantation of a mega-prosthesis can reach 23.5 with a traditional two-stage revision. In our study, the mean MSTS score of all patients was 20 (range 17–23), and the three patients who received the improved revision strategy had scores of 20, 22, and 23, respectively, which is similar to or better than when a conventional two-stage revision is performed.

The primary limitation of this study is the small number of patients included. A larger number of cases with a controlled cohort are needed to further examine the clinical results and associated complication rates of this improved revision strategy.

## Conclusions

In conclusion, total operation time and blood loss during LSS for osteosarcoma are the main risk factors of early PJI after implantation. Combining clinical symptoms and signs with ESR might be a dependable method for diagnosis of PJI early after the primary surgery. For patients with a PJI without confirmed eradiation of microorganisms, a combination of prosthesis implantation and bone cement at the second stage can achieve satisfactory infection control and limb function.

## Data Availability

All data generated or analyzed during this study are included in this published article.

## Abbreviations

PJI: Periprosthetic joint infection
LSS: Limb salvage surgery
ESR: Erythrocyte sedimentation rate
WBC: White blood cell
CRP: C-reactive protein
MSTS: Musculoskeletal tumor society
P. Femur: Proximal femur
D. Femur: Distal femur
P. Tibia: Proximal tibia
PCT: Procalcitonin
